# Prediction of Collision Cross Section Values: Application to Non-Intentionally Added Substance Identification in Food Contact Materials

**DOI:** 10.1021/acs.jafc.1c06989

**Published:** 2022-01-18

**Authors:** Xue-Chao Song, Nicola Dreolin, Tito Damiani, Elena Canellas, Cristina Nerin

**Affiliations:** † Department of Analytical Chemistry, Aragon Institute of Engineering Research I3A, CPS-University of Zaragoza, Maria de Luna 3, 50018 Zaragoza, Spain; ‡ Waters Corporation, Altrincham Road, SK9 4AX Wilmslow, U.K.; § Institute of Organic Chemistry and Biochemistry, Flemingovo náměstí 542/2, 160 00 Prague, Czech Republic

**Keywords:** ion mobility, collision cross section, NIAS, food contact materials, machine learning

## Abstract

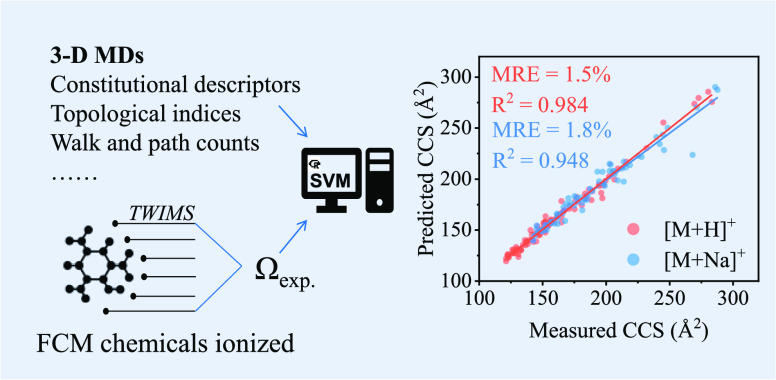

The synthetic chemicals in food contact materials can migrate into food and endanger human health. In this study, the traveling wave collision cross section in nitrogen values of more than 400 chemicals in food contact materials were experimentally derived by traveling wave ion mobility spectrometry. A support vector machine-based collision cross section (CCS) prediction model was developed based on CCS values of food contact chemicals and a series of molecular descriptors. More than 92% of protonated and 81% of sodiated adducts showed a relative deviation below 5%. Median relative errors for protonated and sodiated molecules were 1.50 and 1.82%, respectively. The model was then applied to the structural annotation of oligomers migrating from polyamide adhesives. The identification confidence of 11 oligomers was improved by the direct comparison of the experimental data with the predicted CCS values. Finally, the challenges and opportunities of current machine-learning models on CCS prediction were also discussed.

## Introduction

The food contact materials (FCMs) can provide a protection for food, but meanwhile it is also an important source of contaminations of food. In the manufacturing process of FCM, a range of synthetic additives (antioxidants, plasticizers, photoinitiators, lubricants, slip agents, etc.) are routinely employed to provide the material with desired mechanical and thermal properties. These compounds are intentionally added substances (IAS) and their specific migration limits (SMLs) are included in the positive list of Regulation (EU) No. 10/2011.^1^ On the other hand, non-intentionally added substances (NIAS) are chemicals that are present in a FCM but have not been added for a technical reason during the manufacturing process, and originate from degradation of additives (e.g., 2,4-di-*tert*-butylphenol from Irgafos 168),^2^ interactions between constituents (e.g., 1,6-dioxacyclododecane-7,12-dione from the condensation reaction between 1,4-butanediol and adipic acid),^3^ and impurities of raw materials.^4^ Recycling can also introduce different kinds of NIAS due to the low efficiency of cleaning processes. Oligomers and degradation products can also be produced due to the high temperature and to the presence of oxygen in mechanical recycling.^5,6^ Both IAS and NIAS can migrate through the packaging into food products and have the potential to endanger human health.^7,8^ The risk associated with the migration of NIAS from specific packaging materials has to be assessed.^1^ As the first step of risk assessment, the structural elucidation of such molecules is crucial for the correct quantification and the subsequent toxicological evaluation.

Compared to IAS, the identification of NIAS is much more challenging due to the complexity of composition of the final packaging material and limited ingredient information provided by manufacturers. Gas chromatography–mass spectrometry (GC–MS)^9^ and liquid chromatography–mass spectrometry (LC–MS)^10^ are widely used analytical techniques for the screening of volatile and non-volatile NIAS. A high-resolution mass spectrometer operating in data-independent acquisition (DIA) mode can provide accurate mass of precursor and product ions, thus deriving the elemental composition also based on isotopic pattern distributions. The chemical structure of unknowns can then be inferred from fragmentation studies, applying a combination of common rules. However, in this process, two main issues can be encountered. First, chromatographic coelution exists, which makes it difficult to identify the actual precursor ion, especially where the number of adducts is limited due to different ionization efficiencies. Second, it is possible that two or more candidates conform to the exact mass and a similar fragmentation pathway. In this case, the experience and technical skillfulness of the analyst in the MS spectral interpretation are essential for reducing false detects and to bring confidence to the identification results, which ultimately rely on the confirmation with a pure standard. In this context, the availability of different separation techniques in combination with conventional LC–HRMS systems would be extremely beneficial.

Ion mobility spectrometry (IMS) is a gas-phase separation technique, which enables the separation of ions by collisions with a buffer gas (usually nitrogen or helium) under a defined electric field profile and controlled gas pressure in a drift cell.^11^ The drift time of ions is associated with their size, shape, and charge, which results in a partial orthogonality with MS separation,^12^ besides, the drift time is generally in the range of milliseconds, which fits well between LC separation (in the range of several seconds) and MS detection (on the microsecond scale). The combination of ultrahigh performance liquid chromatography with an ion mobility-mass spectrometry (UHPLC-IMS-MS) can provide a three-dimensional (3D) separation (retention time, drift time, and *m*/*z*), thus increasing peak capacity compared to UHPLC-MS alone.^13^ A few studies reported coelution of isomer pairs in conventional LC, which were then resolved by IMS.^14,15^ In recent years, UHPLC-IMS-MS has been widely used in the structural characterization of lipids,^16^ glycans,^17^ and small molecules, such as pesticides,^18^ steroids,^19^ phenolics,^20,21^ and NIAS in food packaging.^22,23^

Collision cross section (CCS) can be related to the mobility of ions and it is commonly recognized to represent the effective rotationally averaged collision area of the ions with neutral gas molecules, which is a physicochemical property of ions for a given compound. More precisely, CCS describes the momentum transfer between ions and drift gas particles. Therefore, it is considered as a structural property of ionized molecules, which depends on experimental conditions such as drift gas composition, temperature, and reduced field strength (*E*/*N*, where *E* represents the electric field and *N* is the gas number density).^24^ However, unlike drift time, CCS values are not instrument-dependent, so they should be comparable across different instruments and laboratories operating under the same experimental conditions. CCS can then be treated as an additional structural descriptor obtained from IMS for confirmation of compound identification. A number of previous works have demonstrated a fairly good reproducibility of CCS values between different laboratories and platforms.^25,26^ In recent years, several CCS databases have been generated from experimental measurements,^27−32^ but many of them are still difficult to integrate into routine discovery analyses. In addition, unless costly and time-consuming chemical synthesis and purifications of suspect compounds are addressed, the empirical CCS values of compounds cannot be obtained when their standards are not commercially available. In order to enhance the wider application of CCS in qualitative analysis, a number of efforts have been made in the past few years for the prediction of a compound’s CCS from its molecular descriptors (MDs) (i.e., numeric values that provide a fingerprint of a compound’s structural and physicochemical properties) by means of machine-learning tools.^18,20,33−36^ Different algorithms, such as partial least squares regression (PLS-R), support vector regression (SVR), and artificial neural network (ANN), have been applied to create predictive models for specific groups of analytes. The number of MDs used to develop the predictive models varies from tens to thousands. As an alternative to MDs, Ross and co-workers used molecular quantum numbers (MQNs), which are obtained from analyzing compounds as a molecular graph (i.e., collections of nodes = atoms, and edges = bonds), claiming that MQNs are invariant with respect to the software used to compute them.^37^ Plante and collaborators developed a convolutional neural network model (CNN) using simplified molecular-input line-entry systems (SMILESs) as the input for CCS prediction, without the need for MDs.^34^ When no CCS database or commercial standards are available, the machine-learning approach can become a potential alternative to predict and confirm CCS values.

In this study, a traveling wave collision cross section in nitrogen (^TW^CCS_N2_) library was generated by measuring 488 standards available in our laboratory via UPLC-IMS-QToF. The majority of the measured compounds are commonly used chemicals in food-packaging materials. The chemical structures of these compounds were then submitted to dedicated software to retrieve the physicochemical descriptors. The goal was to develop an *in-house* prediction model to predict ^TW^CCS_N2_ values of specific compounds using MDs as the input. After optimization and comparison with the currently available tools, the developed predictive model was implemented within our NIAS identification pipeline, and employed for the structural elucidation of unknown compounds migrating from packaging materials. Finally, we provide a discussion on the challenges and opportunities of existing machine-learning CCS prediction tools.

## Materials and Methods

### Chemicals and Reagents

A total of 488 standards, including the commonly used additives in food packaging, such as antioxidants, plasticizers, dyes, slip agents, UV-absorbers, lubricants, as well as a large set of NIAS historically found from our previous studies (degradation products of hindered phenolic antioxidants, oligomers, by-reaction products, etc.) were included in the dataset. All standards were purchased from Sigma-Aldrich Quimica S.A. (Madrid, Spain), Extrasynthese (Genay, France), and Cayman chemical company (Ann Arbor, Michigan, USA). HPLC grade methanol (≥99.9%), ethanol (≥99.9%), dichloromethane (≥99.8%), and dimethyl sulfoxide (≥99.8%) were purchased from Scharlau Chemie S.A (Sentmenat, Spain). Ultrapure water was produced using a Millipore Milli-QPLUS 185 system (Madrid, Spain). Formic acid was purchased from Waters (Milford, MA, USA). For building the CCS database, standard stock solutions (1000 mg kg^–1^) were prepared by dissolving 10 mg of standards in 10 g of methanol. Other solvents, such as ethanol, dichloromethane, and dimethyl sulfoxide were used when the standards were not dissolved in methanol. The stock solutions were then diluted to create working solutions at ∼1 mg kg^–1^. Each working solution contained 8–10 analytes, avoiding isomers and coeluting compounds in the same mixture. All standard solutions were stored in the dark at −20 °C until analysis.

### Measurements of Experimental CCS Values

For the empirical measurements of ^TW^CCS_N2_ values, an Acquity I-Class UPLC system coupled to a Vion IMS-QToF mass spectrometer (Waters, Manchester, UK) was used. UPLC separation was performed on a CORTECS C_18_ column (2.1 × 100 mm, 1.6 μm particle size, 90 Å pore size) at a flow rate of 0.3 mL min^–1^. The column temperature was 40 °C. The mobile phase was composed of water (A) and methanol (B), both with 0.1% of formic acid (v/v). The volumetric percentage of mobile phase B during the LC gradient was as follows: 0–7 min: 5–100%; 7–11 min: 100%; 11–11.10 min: 100–5%; and 11.10–13 min 5%.

The Vion IMS-QToF [IMS resolution ∼20 Ω/ΔΩ full width at half-maximum (fwhm)] consists of hybrid quadrupole orthogonal acceleration time-of-flight mass spectrometers, in which a stacked-ring ion guide, that is, the mobility cell, is positioned before the quadrupole mass filter. The system was operating in positive electrospray mode (ESI+). The capillary voltage was 1 kV and sampling cone voltage was 30 V, the source temperature was 120 °C, cone gas flow was 50 L h^–1^, and N_2_ was used as a desolvation gas with a flow rate of 800 L h^–1^ at 500 °C. Mass and CCS calibration were performed in the range 50–1200 *m*/*z* and 130.4–372.6 Å^2^, respectively, using the Major Mix IMS/ToF Calibration Kit (Waters Corp.). LockSpray containing Leucine-Enkephalin ([M + H]^+^, *m*/*z* 556.2771) at a concentration of 100 ng mL^–1^ and an infusion rate of 15 μL min^–1^ was used for real-time mass correction. Raw data were acquired in high-definition MS^E^ mode (HDMS^E^), and the mass spectra were acquired with an acquisition rate of 0.2 s at two collision energy states (low energy = 6 eV, and high energy ramp = from 20 to 40 eV). Nitrogen was used as a drift gas and argon was used as a collision-induced dissociation (CID) gas. The ToF analyzer was operated in sensitivity mode, and the ion mobility settings were as follows: an IMS gas flow rate of 25 mL min^–1^, a wave velocity of 250 m s^–1^, and an IMS pulse height of 45 V. Data acquisition and processing were carried out on UNIFI v.1.9 software (Waters Corp.).

Prior to each analysis, an in-house made Test-Mix solution was injected for a system suitability test. The molecular formula, monoisotopic mass, and expected CCS of nine compounds in Test-Mix are listed in Table S1. The pass/fail criteria for mass and CCS accuracy were: mass error <5 ppm and ΔCCS <2%. All working solutions were injected in triplicate, with an injection volume of 5 and 10 μL, for a total of six technical replicates per each compound. This allowed an easier assignment of standard peaks and higher confidence in the experimental ^TW^CCS_N2_ values, which were obtained by averaging *n* = 6 independent measurements.

### CCS Prediction

Multivariate PLS is one of the most widely used machine-learning algorithms for both regression and classification purposes; its basic knowledge can be found in the literature.^38^ Support vector machine (SVM) is a supervised learning algorithm that can be used for both classification and regression analysis, and it has been used for CCS prediction in previous studies.^36,39^ Herein, both PLS and SVM models were developed between the physicochemical MDs of all the compounds and their experimentally derived ^TW^CCS_N2_. MDs were obtained using alvaDesc software v.2.0.4 within the Online Chemical database (OCHEM, http://ochem.eu/home/show.do), obtaining a total of 5666 MD. The detailed list of the generated descriptors is reported in Table S2.

The irrelevant descriptors were eliminated before the model building. The descriptors with constant values or with very few unique values relative to the number of samples contain few information, which were considered less important for the CCS prediction. These kinds of descriptors were removed by function of *nearZeroVar* in R package *caret*.

The dataset was randomly split into training and testing sets in a 3:1 ratio. By doing so, the prediction ability of a developed model can be assessed in an unbiased manner. Descriptive statistics (i.e., mean, standard deviation, range, and median) of [M + H]^+^ and [M + Na]^+^ adducts’ CCS for both calibration and validation sets are summarized in Table S3, and Figure S1 shows the distribution of data points in calibration and validation sets.

Prior to modeling, natural logarithm transformation was applied to ^TW^CCS_N2_ values to promote data normality (Figure S2). The MD data (training set) were mean-centered and scaled to unit variance using the following equation
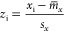
where *z*_i_ is the normalized data for the variable *x* of a particular molecule i; *m̅*_*x*_ and *s*_*x*_ are the mean and standard deviation of *x*. The *m̅*_*x*_ and *s*_*x*_ computed for the training set were then used as normalization factors for the testing set. Both models were built on the preprocessed (training) data and optimized through 10-folded cross validation. The number of latent vectors of PLS was optimized based on the root mean squared error of cross validation (RMSECV) and prediction residuals, and both statistically inspired modification of the partial least squares (SIMPLS) and kernel PLS were used to build the model. As for SVM, two hyperparameters were optimized in order to get an accurate prediction: cost of constraints violation (*C*) and gamma (γ). Eight groups of *C* values (0.001, 0.005, 0.01, 0.025, 0.05, 0.1, 0.25, and 0.5)/*N*_MD_ (i.e., the number of MDs) and nine γ values (2^0^ to 2^8^) formed 72 parameter combinations. The parameter combination providing the minimum RMSECV was used for further SVM model.

Sensitivity ratio (SR) is an embedded method within PLS-R for evaluating the contributions of variables for the model, which is defined as the ratio between the explained and the residual variance in the target-projected component.^40^ The *F*-test (99% confidence interval) criterion was used to define the boundary between highly important and less-important variables, as proposed by Rajalahti et al. (2009).^40^ The important descriptors were then to build PLS and SVM models and their performances were compared with models built without feature selection.

Four CCS prediction models were developed for each adduct based on two algorithms (PLS and SVM) and two types of MDs (all MDS and important MDs selected by SR). The CCS values of the testing set were predicted with four models obtained above. The prediction results of the model with a better performance were then compared with the three main CCS prediction tools currently available, which use either MDs or MQNs: AllCCS from Zhu Lab,^39^ CCSbase from Libin Xu Lab,^37^ and CCSondemand from Broeckling and co-workers.^41^

All data processes and calculations were performed in R (version 4.0.5) using internal statistical functions and external packages (i.e., *pls* for PLS-based prediction, *e1071* for SVM-based model, *plsVarSel* for feature selection, and *ggplot2* for plot creation).^42^

### Sample Preparation and Extraction

The CCS predictive model was applied to the identification of NIAS in water-based adhesives, polyamide 6 (PA6) and polyamide 66 (PA66). Previous studies suggested that cyclic oligomers can be present in these types of materials.^3,43^ For the extraction of oligomers from adhesives, 5 g of sample was mixed with 50 mL of water, the mixture was centrifuged at 4000 g for 10 min, and the supernatant was passed through a hydrophilic–lipophilic balance copolymer SPE (Oasis HLB cartridge, 6cc, Waters Corp.), previously activated with 10 mL of methanol and 10 mL of water. The oligomers were eluted with 50 mL of methanol and analyzed via LC-IMS-HRMS. For the extraction of oligomers from PA6 and PA66, 10 g of pellets was extracted with 50 mL of methanol at 40 °C overnight, the solution was filtered using a 0.22 μm nylon membrane filter and the filtrate was evaporated using a rotary evaporator. The residue was redissolved in 10 mL of 10% methanol in water (v/v). The reconstituted extract was cleaned up on SPE and analyzed following the procedure described above.

As the commercial standards of these oligomers were not available, these were attempted to be produced at the laboratory scale to verify the identification. Briefly, 1 g of adipic acid was mixed with 1 g of 1,4-butanediol in a melting crucible with a lid (40 mL), the mixture was heated at 135 °C for 2 h, the obtained liquid was dissolved in methanol at a concentration of 10 mg kg^–1^, and then analyzed by LC-IMS-QToF under the conditions described in the Experimental Section.

## Results and Discussion

### Mass-to-Charge and CCS Correlation

**Figure 1:**
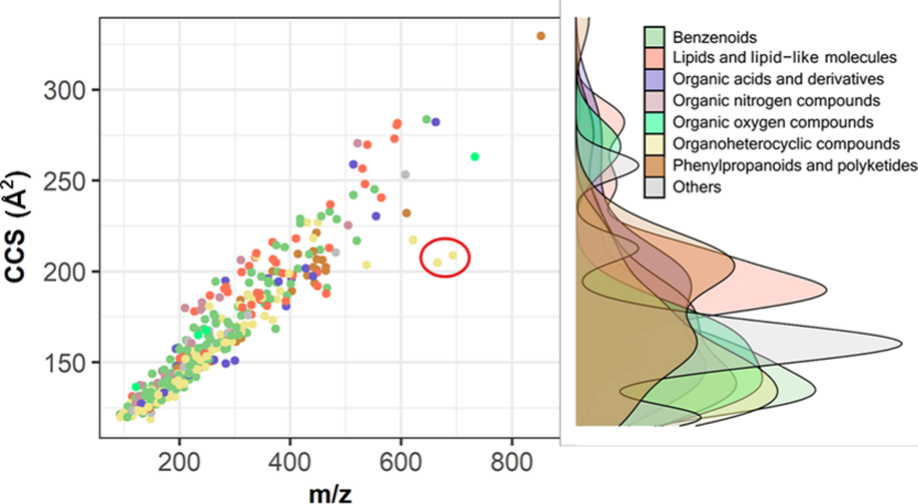
Correlation between mass-to-charge and CCS of [M + H]^+^ adducts and its CCS density distributions according to the compounds’ chemical class. The red circle highlights bromophenol blue and bromocresol green, which significantly deviate from the mass/CCS trend.

A total of 635 ions (i.e., 380 [M + H]^+^ and 255 [M + Na]^+^ adducts) were detected for the 488 analyzed standards, with ^TW^CCS_N2_ values ranging from 118.6 to 329.4 Å^2^, whose distribution is shown in Figure 1. As expected, a significant correlation (*R*^2^ = 0.880 and 0.878 for [M + H]^+^ and [M + Na]^+^, respectively) was found between the CCS and the respective ion *m*/*z*. Interestingly, lower *R*^2^ were observed in the present work with respect to similar previous studies, which focused on specific compound classes characterized by recurring subunits/structures (e.g., phenolic compounds, peptides).^20,44^ In fact, the standard analyzed in this work contained several types of small molecules: carbonyls, organic acids, esters, and amides; including alkyl and aryl moieties, typical of some classes of additives (see Figure 1), and the chemical classes of analyzed standards were obtained from ClassyFire^45^ and shown in Figure S3. Benzenoids, lipids and lipid-like molecules, and organoheterocyclic compounds seem to be the major classes, and some additives: phthalate-based plasticizers, antioxidants, bisphenols, and primary aromatic amines belong to benzenoids.

CCS and the mass-to-charge ratio for both protonated and sodiated molecules presented 12% orthogonality (*O*),^41^ which was calculated using
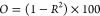
where *R*^2^ is the Pearson determination coefficient of the linear regression. This suggests that the inclusion of the CCS into a compound elucidation workflow for E&L testing could potentially increase peak capacity of >10% compared to the retention time and accurate mass alone; this could ultimately increase the number of detected and identified analytes. Similar observations were made in the metabolomics context by several authors.^46,47^

Molecular mass was not the only descriptor affecting the CCS values; two compounds significantly deviated from the mass/CCS trend. These were bromophenol blue and bromocresol green, dyes used in the packaging industry and as pH indicators. In addition to C, H, O, and N, these compounds include bromine (Br) within their structure, which could be ascribed to the observed negative deviation in the correlation plot.

For most of the analyzed molecules, [M + Na]^+^ showed higher CCS values compared to [M + H]^+^, as expected, due to the higher atomic radius and mass of Na over H. However, in some cases, the CCS values for [M + H]^+^ were higher than those for [M + Na]^+^. For example, bis(2,4-dicumylphenoxy) pentaerythritol diphosphite, a common antioxidant used in food packaging, presented ^TW^CCS_N2_ values of 329.4 and 311.4 Å^2^ for its [M + H]^+^ and [M + Na]^+^ adducts, respectively. The Na^+^ ion can be embedded in the intricate structure of the molecule, which can easily rotate and bring about diverse conformation in the 3D space. The sodium can be trapped in the core of the molecule and the proton might be protruding from one side of the molecule, thus resulting in the protonated adduct to be larger in size compared to the sodiated adduct.

### Charge Isomers

**Figure 2:**
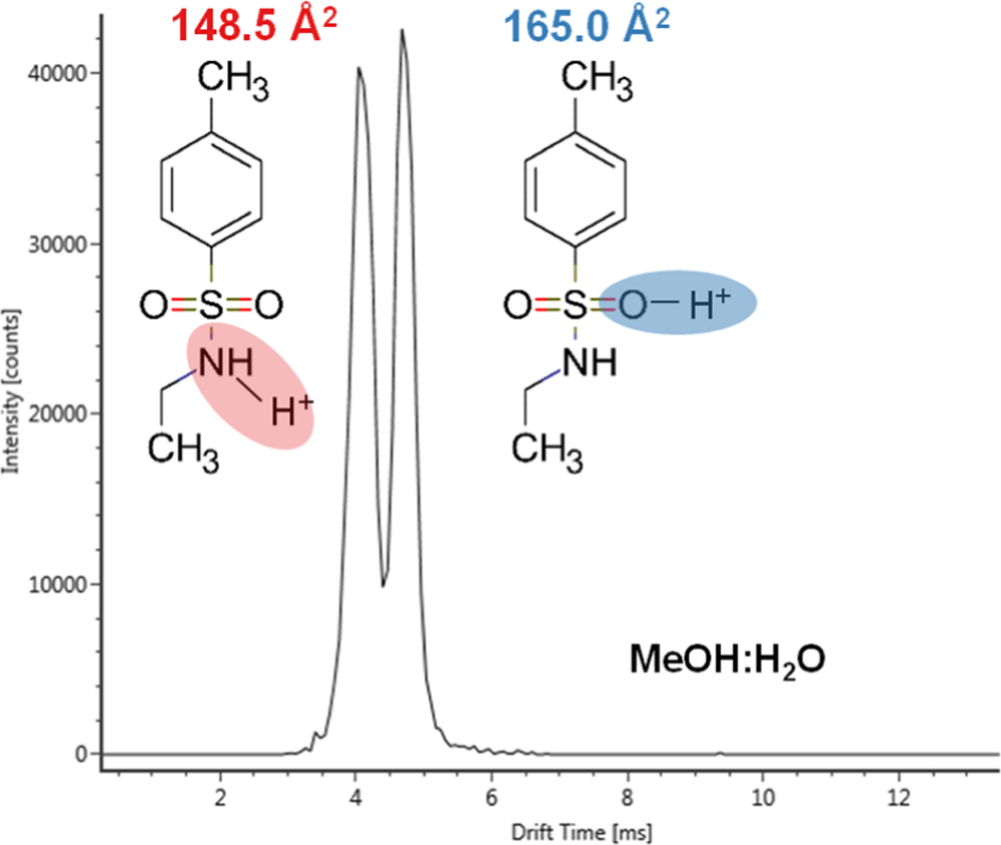
ATD of *N*-ethyl-*p*-toluenesulfonamide (*m*/*z* 199.0667) and its possible charge isomers in the methanol/water mobile phase.

In some cases, certain compounds can adopt multiple gas-phase conformers, resulting in multiple Gaussian-shaped arrival time distributions (ATD). In ESI+, this is commonly due to the presence of multiple equivalent protonation sites on the neutral molecule (giving rise to protomers), as well as multiple stable conformers from a single protonation site. If a charge isomer pair is sufficiently resolved in the IM dimension, the peak-detection algorithm will recognize two different components and will assign two discrete CCS values. The relationship between the charge location and the experimental CCS is logical, as the location of the charge affects the three-dimensional conformation of an ion, thus the CCS will be affected too. *N*-Ethyl-*p*-toluenesulfonamide, a commonly used plasticizer in polyamides and cellulose acetate materials, showed two ^TW^CCS_N2_ values for its [M + H]^+^ adduct. As shown in Figure 2, protonation might occur on both O and N, leading to two different charge isomers, characterized by a double peak in the ATD of this compound, therefore leading to different CCS. Interestingly, by replacing methanol with acetonitrile as the organic mobile phase, the formation of more compact conformation is favored (ATD peak at 4.15 ms over peak at 4.83 ms, Figure S4). Warnke and co-workers found that aprotic solvents can facilitate the protonation of amines, whereas methanol/water facilitate the protonation on carbonyl oxygen.^48^ This led us to speculate that the first species (4.15 ms) corresponded to the protonation of the nitrogen atom, forming the quaternary ammonium cation, while the second species (4.83 ms) was represented by the protonation of the oxygen atom.

The relatively unpredictable formation of charge isomers and, generally speaking, conformers, represents a great challenge when attempting to create a CCS database and to apply prediction models. Essentially, the MDs for such isomers will likely be identical, regardless of the reference MD library of choice; thus, the prediction algorithm will be unable to generate multiple outputs for the isomeric adducts.

### Dimeric Ionic Species

In some instances, the presence of two (or more) features for the same precursor ion can be due to the formation of dimers, trimers, or other non-covalent clusters in the ESI source, which will be subjected to a change in conformation or chemical reaction while traveling through the mobility cell (e.g., gas-phase collisional ion activation) or at a later stage within the ion path.^49^

For example, 12-aminododecanolactam is a cyclic monomer of polyamide 12; the mobility trace of its sodiated precursor ions is shown in Figure S5. Two distinct peaks were observed at 4.79 and 6.74 ms. Besides these two main peaks, a less-intense peak at 5.78 ms was also discerned, corresponding to a CCS value of 191.3 Å^2^. It can be speculated that the dimer precursor ion [2M + Na]^+^ undergoes a fragmentation process to [M + Na]^+^ during its permanence time in the drift region, leading to two different peaks in the ATD, with an additional peak that could correspond to a transition-state species or an artifact. Another example is tributyl phosphate, a commonly used defoamer and plasticizer, which formed [2M + H]^+^ and [2M + Na]^+^ in the ESI source. A portion of these ions could have fragmented to [M + H]^+^ and [M + Na]^+^ after passing through the drift cell or while transiting through the ion guides, thus leading to two distinct peaks in the mobility trace (Figure S6).

### Development and Optimization of the CCS Prediction Method

After the elimination of non-informative descriptors, the final number of retained descriptors was *n* = 3036. Four models were developed for the [M + H]^+^ and [M + Na]^+^ adducts, respectively. Through the feature selection by SR, 1029 and 862 descriptors were selected for [M + H]^+^ and [M + Na]^+^ adducts, respectively.

The performances of the final PLS and SVM models are summarized in Table S4 and the violin-plot of prediction errors are shown in Figure S7. Overall remarkable prediction performance was achieved for [M + H]^+^, regardless of the considered MD set. Furthermore, it is worth stressing that any issue related to data overfitting can be diagnosed and excluded as the prediction ability was assessed by external validation. KernelPLS can deal with non-linear behavior. For [M + H]^+^, KernelPLS shows slight improvements in the prediction accuracy compared to SIMPLS, but is still less accurate than SVM. For [M + Na]^+^, KernelPLS does not show a significant difference with SIMPLS. A slightly better prediction accuracy was achieved by SVM for both [M + H]^+^ and [M + Na]^+^, 62.1 and 54.7% of compounds in [M + H]^+^ and [M + Na]^+^ were predicted with <2% errors. The prediction of CCS was less accurate along with the feature selection, as can be seen in Figure S7 and Table S4; in SVM-based models, the proportions of compounds with <2% predicted errors decreased from 62.1 to 53.7%, from 54.7 to 48.4%, for [M + H]^+^ and [M + Na]^+^, respectively. For these reasons, we refer to SVM and 3036 descriptors for the prediction of CCS of [M + H]^+^ and [M + Na]^+^.

The first 25 important descriptors for the prediction of CCS are shown in Figure S8. The Hosoya-like index (log function) from the Barysz matrix weighted by Sanderson electronegativity (Ho_Dz.e.), the Hosoya-like index (log function) from the Barysz matrix weighted by ionization potential (Ho_Dz.i.), McGowan volume (Vx), van der Waals volume from the McGowan volume (VvdwMG), and sum of atomic Van der Waals volumes (Sv) were the five most important descriptors for the prediction of CCS of [M + H]^+^. Ho_Dz.e. was used to predict CCS of [M – H]^−^ previously.^50^ Other types of important MDs were 2D matrix-based descriptors, such as the spectral moment of order 3 from the Barysz matrix weighted by Sanderson electronegativity (SM3_Dz.e.) and Hosoya-like index (log function) from the topological distance matrix (Ho_D), these MDs were also used to predict CCS values.^35,50^ Sum of atomic polarizabilities (Sp) and Ghose–Crippen molar refractivity (AMR) were another two important MDs, which were used in CCS prediction in Zhou et al. (2016).^36^

**Figure 3:**
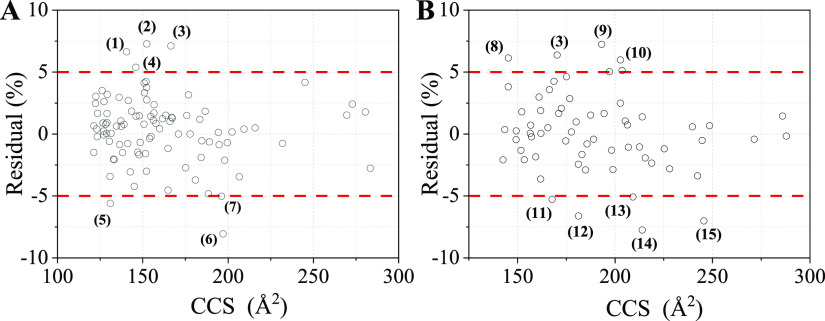
Residuals % (percentage relative prediction error) of the external validation set for [M + H]^+^ (A), and [M + Na]^+^ (B) adducts: (1) 4,4′-difluorobenzophenone; (2) 4-aminophenyl sulfone; (3) Tebuconazole; (4) 2,2′,6′,2″-terpyridine; (5) phenyl isothiocyanate; (6) diphenyl(2,4,6-trimethylbenzoyl)phosphine oxide; (7) 4-(dodecyloxy)benzoic acid; (8) 1,4,7-trioxacyclotridecane-8,13-dione; (9) dehydrocholic acid; (10) tetracycline; (11) dibutyl phosphate; (12) triphenylphosphine oxide; (13) corticosterone; (14) testosterone propionate; and (15) rutin.

Relative prediction residuals of validation set are shown in Figure 3. When comparing the [M + H]^+^ and [M + Na]^+^ models, the former showed a better predictive performance, 92.6% (88/95), of protonated molecules showing prediction errors less than ±5%; for [M + Na]^+^, only 81.3% (52/64) of molecules were predicted with ±5% error. This phenomenon can possibly be due to the fact that MDs were calculated on the neutral form of the molecules. The sodium ion has a higher atomic radius compared with a proton, thus the descriptors of sodium adducts could differ significantly compared to the descriptors of the neutral molecules. This observation is in accordance with the findings from Bijlsma et al., where the author obtained lower prediction errors for [M + H]^+^ compared to [M + Na]^+^ species.^18^

### Detection of Outliers

The molecules with prediction errors higher than ±5% (outliers) were further investigated to try to understand the cause of poor prediction. The measured and predicted ^TW^CCS_N2_ of sodiated (−)-erythromycin were 268.2 and 223.6 Å^2^, respectively (a prediction deviation of −16.6%). The high prediction error of this compound could be a consequence of the fact that only three compounds with CCS values higher than 280 Å^2^ were present in the calibration set. The limited training data points in the high-end CCS range could bias the prediction. In addition, some compounds containing halogens (fluorine, chlorine, and bromine) also showed relatively high prediction errors. Tebuconazole, a commonly used triazole fungicide, and its protonation ion had prediction error of 7.1% (measured 164.8 Å^2^, predicted 178.3 Å^2^). Bisacylphosphine oxides, a commonly used UV photoinitiator in packaging, containing phosphorus, also presented a high prediction error of 5.1% (measured 203.6 Å^2^, predicted 214.1 Å^2^). The presence of these outliers may be due to the fact that most compounds in the data set prevalently contained C, H, O, and N; only a few compounds contained halogens and P. This highlights the importance of the chemical class when considering such tools. To further improve the model, the incorporation of more compounds with diverse chemical structures, especially the compounds with high molecular mass and with less-common elements, such as halogens and phosphorus, should be considered.

### Comparison of the Herein-Developed SVM Model with Existing CCS Predicting Tools

**Figure 4:**
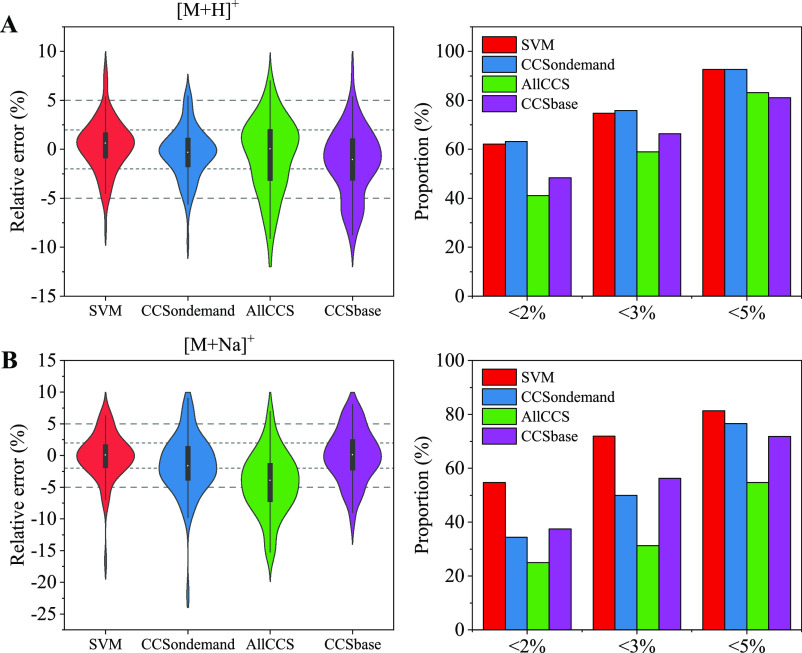
Violin-plots and bar-plots showing the comparison between the developed SVM model with other predicting tools. [M + H]^+^ (A) and [M + Na]^+^ (B) adducts.

CCSondemand is a recently developed CCS prediction tool, which is based on the gradient boosting (GB) algorithm and 3775 ^TW^CCS_N2_ data of different chemical classes.^41^ AllCCS is based on the SVR algorithm and more than 5000 experimental CCS records,^39^ and CCSbase is a web interface that breaks down the chemical structural diversity by unsupervised clustering, followed by training of specific prediction models on each cluster.^37^ The comparison of CCS prediction of the validation set between our SVM model with these three CCS predicting tools is illustrated in Figure 4. Table S5 shows the detailed predictive performance indices of the tested models.

As can be seen, the herein developed model presented a better predictive performance for both [M + H]^+^ and [M + Na]^+^ compared to other tools: 92.6% (88/95) of protonated molecules and 81.3% (52/64) sodiated molecules showed prediction error less than 5%. CCSondemand showed a comparable predictive ability for [M + H]^+^ and a slightly worse predictive ability for [M + Na]^+^. This is understandable because the dataset used for the development of CCSondemand includes a portion of experimental ^TW^CCS_N2_ data of chemicals in food packaging. AllCCS and CCSbase showed less-accurate predictions, where 83.2 and 81.1% of [M + H]^+^ were predicted with <5% error, respectively. Some compounds typically used as food-packaging additives were predicted with high errors: for 3,5-di-*tert*-butyl-4-hydroxybenzaldehyde, a degradation product of butylated hydroxytoluene (BHT)^51^ with the measured ^TW^CCS_N2_ of its [M + H]^+^ is 164.9 Å^2^, and AllCCS gave a predicted CCS of 149.8 Å^2^ (−9.1%) and CCSbase gave a predicted CCS of 157.1 Å^2^ (−4.7%). Some primary aromatic amines also presented high prediction error by AllCCS and CCSbase, such as 4-aminobiphenyl (−5.8% and −6.4, respectively), benzidine (−6.3 and −8.0%, respectively), and 2,6-dimethylaniline (−6.0% and −5.6, respectively). Additionally, CCSbase gave a high prediction error for aniline (−5.2%), 4-chloroaniline (−8.0%), 4-chloro-2-methylaniline (−6.3%), and 5-chloro-2-methylaniline (−6.1%). The relatively less-accurate CCS prediction of these kinds of compounds by AllCCS and CCSbase was possibly due to the fact that the molecules used for model training do not exhibit the similar structural characteristics to the chemicals in food packaging, and the quality of prediction is notably affected by the types of molecules used for training.^39^ Another thing needed to be mentioned is that even though the SVM model herein showed a better CCS predictive performance than AllCCS and CCSbase for the chemicals in food packaging, the more diverse chemical classes of AllCCS and CCSbase cannot be ignored, and these two prediction tools can be applied to a wide variety of molecules.

### Application of SVM to NIAS Identification

The applicability of the developed CCS prediction model to the NIAS identification was further assessed by the analysis of a series of oligomers from adhesives and polyamides (PAs). Oligomers are molecules that consist of identical repeating units, which can be formed by the incomplete polymerization of monomers during polymer manufacturing and also due to the polymer degradation process.^6^ Based on previous knowledge of the composition of adhesives and PAs,^3,43^ 12 oligomers were tentatively identified through suspect screening. Four adhesive oligomers were derived from the reaction between adipic acid and 1,4-butanediol, five PA6 oligomers originated from the polymerization of caprolactam, and three PA66 oligomers were derived from the reaction of 1,6-diaminohexane and adipic acid. The detailed comparison between the experimental and predicted CCS of oligomers is shown in Table S6. For 11 compounds, the confidence of the structural elucidation process was improved by considering the predicted ^TW^CCS_N2_ values within the workflow. The oligomers with low and high mass tend to present higher prediction errors, such as 1,8-diazacyclotetradecane-2,9-dione and 1,6,13,18,25,30,37,42-octaoxacyclooctatetracontane-7,12,19,24,31,36,43,48-octaone. This also emphasized the importance of incorporating more high-mass and low-mass molecules in the training set. The mass spectral and fragment assignment of 1,6,13,18,25,30-hexaoxacyclohexatriacontane-7,12,19,24,31, 36-hexone is shown in Figure S9.

Two cyclic oligomers were found by suspect screening of the reaction products between adipic acid and 1,4-butanediol, which showed the same RT and ^TW^CCS_N2_ with the compounds identified in water-based adhesive: 1,6,13,18-tetraoxacyclotetracosane-7,12,19,24-tetrone (5.83 min), [M + H]^+^ (189.1 Å^2^), [M + Na]^+^ (188.0 Å^2^), and [M + NH_4_]^+^ (190.9 Å^2^); 1,6,13,18,25,30-hexaoxacyclohexatriacontane-7,12,19,24,31,36-hexone (6.47 min), [M + H]^+^ (232.8 Å^2^), [M + Na]^+^ (228.7 Å^2^), and [M + NH_4_]^+^ (238.3 Å^2^). These data were in accordance with the prediction outcomes and further proved the reliability of identification.

### Challenges and Opportunities of Existing Machine-Learning CCS Prediction Models

Charge isomers, dimers, chiral ions, and IMS resolving power: in the previous section, we have seen that small molecules can give rise to different charge isomers (e.g., protomers) and dimers. In both cases, multiple or distorted peaks in the ATD are obtained, which, when sufficiently resolved, are associated with multiple CCS values. Because current ML algorithms return a single CCS value for each compound as the output data, these algorithms do not take into account the presence of charge isomers or chiral ions. This leads to potential incorrect predictions. In addition, conformers are often not fully resolved due to the relatively low resolving power of the existing IMS-MS systems [typically Rp < 60 fwhm for linear temporally dispersive IMS devices, such as traveling wave ion mobility spectrometer and drift tube ion mobility spectrometer.^52^ Fortunately, technological development is on-going, and recent (or soon) commercially available platforms such as cyclic ion mobility (cIMS)^53^ and structures for lossless ion manipulations (SLIM)^54^ are expected to provide a higher IMS resolving power, thus potentially a better resolution of conformers.

The quality of the input data contributes to a good prediction outcome. Perhaps, we should dedicate more effort in the derivation of more accurate experimental CCS from instrumental analysis. So far, it is inappropriate to claim a prediction tool able to reach less than 2% CCS prediction relative error, as the current commercially available ion mobility platforms are set to produce CCS with deviations of ∼1–2% from standard values. For secondary IMS methods [i.e., traveling wave ion mobility spectrometry (TWIMS), thermal ionization mass spectrometry, and drift tube ion mobility spectrometry (DTIMS) operating via a single-field method], the set of standards used as CCS calibrants should be specified. This is particularly important for TWIMS, as different compound classes used as calibrants can have an impact on the derivation of ^TW^CCS.^55^ Recently, Richardson and collaborators revisited the theory of T-wave IMS^56^ and proposed a more precise and robust calibration approach,^57^ which will likely be adopted by next-generation TWIMS systems, and can further improve the experimental ^TW^CCS values as input data.

MDs are mathematical representations of a compound calculated by well-specified algorithms, which transform molecular structures into numbers.^50^ MDs are used as X-block in SVM and represent the second group of the input data for model training in all MD-based machine-learning algorithms. It is, therefore, crucial to obtain accurate MDs for reliable predictions. In the present work, as well as in most of the previously described studies, researchers make use of 2D-MDs calculated for the neutral form of the molecule. This tendency is not strictly correct, as the measured CCS is actually derived for the ionized form of compounds (i.e., adducts). Gonzales and co-workers developed multiple ML prediction models for a group of deprotonated phenolic compounds (training *n* = 56, validation *n* = 16) using 3D-MDs after considering the proton removal from all possible titratable regions, followed by energy reminimization, and considering the most stable conformers.^20^ The authors emphasized the ease of integration of their ML models in metabolite identification, compared to computational chemistry techniques (i.e., Mobcal). Yet, the generalization of Gonzales’ method to a wider range of analytes and adduct types is not straightforward. When considering the 3D conformation of an ion, the first challenge is to assign the location of the charge. We have seen that not only the charge could reside on multiple discrete positions (i.e., charge isomers) but also some compounds can distribute the charge across the molecule (i.e., the mesomeric effect due to the presence of conjugated bonds and aromatic structures). Furthermore, some compounds present dynamic conformations, which means that the transition from one energy-state to another could take place within the millisecond time frame, leading to splitting ATD peaks, sometimes ascribed to artifacts. Last but not least, also the ESI capillary temperature, voltage, and the source pressure can affect the internal energy distribution of electrosprayed ions, which in turn can affect the initial conformation of such species at the ionization stage.^58^ Factoring all these parameters into a prediction model becomes extremely complicated. A potential solution could be to integrate molecular modeling within the ML-prediction workflow in an automatic fashion, so that the user would be only requested to specify linear notation (e.g., SMILES) and adduct type into a script that automatically retrieves all possible ionic conformations, calculates 3D-MDs of the most stable ionic conformers, and uses such refined descriptors as the input data for CCS prediction. The process of encoding refined ionic 3D-MDs as the input features should be performed in a computationally cheap and easy-to-use manner, otherwise such prediction models would remain a tool for privileged users, not applicable to real-life identification workflows. Some authors used 3D-MDs of the neutral molecules, for example, Soper-Hopper and co-workers compared the prediction performance using 2D versus 3D MDs.^50^ They came to the conclusion that only in a few cases 3D models produced predictions better than 2D models, obtaining a RMSE of 7.0 Å^2^ (median error of 2%) using 2D-MDs. However, such a performance could be further improved when considering 3D-MDs of the ionic species. Nevertheless, regardless of the discussion around 2D versus 3D, mining of MDs remains highly customizable (i.e., different MD libraries and tools exist) and it is prone to user error. Thus, an efficient and standardized method for retrieving MDs should be pursued and agreed within the scientific community.

#### Model Universality

Nowadays, CCS prediction models tend to be built on a wider group of training data (e.g., Zhou et al. presented a model trained on more than 5000 experimental values),^39^ including a growing number of compounds and a mix of many different chemical classes. On the other hand, a different approach is to train ML algorithms on specific classes of compounds and to apply such prediction tools for specific applications. In the present work, we demonstrated that the herein developed tool can outperform universal models for the prediction of chemicals in migration assessments from packaging materials. Nevertheless, the benefits of universal models should be acknowledged, as they can be used for all applications, regardless of the compound class.

#### Drift Tube versus Traveling Wave IMS

The most recent and comprehensive ML-based CCS prediction models also merged both ^DT^CCS and ^TW^CCS in both training and validation sets. This would further enhance the universality of such models. However, the fundamental difference of the drift tube and traveling wave technology should not be neglected. Hinnenkamp et al. performed a study in which the CCS of 124 different small molecules were measured on both DTIMS and TWIMS.^26^ The authors found deviations <1% for most substances, but some compounds showed deviations up to 6.2%, which indicates that CCS databases cannot be used without care independently from the instrument type. Plante and co-workers noticed a decline of prediction performance of their CNN model based on a global testing set when considering only the Astarita dataset based on ^TW^CCS [averaged *R*^2^ from 0.97 to less than 0.9, and median relative error from <2.6% to 5%].^34^ The authors hypothesized that a bias in measurement between data sets can be present.

Unknown annotation is one of the major bottlenecks in untargeted E&L analysis. To accelerate the workflow from raw data processing to compound identification, multifactor authentication with the integration of predicted CCS in combination with retention time, accurate mass, and in-silico MS/MS tools can facilitate this challenging task. In this study, we developed a reliable ^TW^CCS_N2_ prediction tool for chemicals in FCMs based on SVM. For more than 90% of protonated molecules, the model accurately predicted CCS with relative errors below ±5%. The SVM model was successfully applied to the analysis of oligomers migrating from FCMs and adhesives, and it was integrated within our suspect and non-targeted analysis workflows for compound discovery and chemical migration assessment. The incorporation of a wider number of compounds in the training set, as well as the employment of a more accurate set of 3D-MDs based on energetically minimized ion species could be explored to enhance model coverage and accuracy. Nevertheless, we believe that an automatic and universal approach for gathering the appropriate MDs from ionized species, also considering charge isomers, can be a game-changer in the prediction of CCS and it should be pursued in order to turn *in-house* prediction models into tools truly applicable in all laboratories.

## Supporting Information Available

Nine compounds in Test-Mix; MDs calculated by alvaDesc; distribution of ^TW^CCS_N2_; performance of PLS-R and SVM; comparing SVM with other tools; predicted CCS values of oligomers; distribution of training and testing sets; distribution of natural logarithms of CCS; chemical classes of compounds; *N*-ethyl-*p*-toluenesulfonamide charge isomers; universal force field energy plot; ion mobility of 12-aminododecanolactam; mass spectra and mobility trace of tributyl phosphate; prediction performance of PLS and SVM; first 25 important descriptors; and identification of adhesive oligomer (PDF)
Empirical CCS database for 635 ions of 488 compounds (XLSX)

## Abbreviations

TWIMS: traveling wave ion mobility spectrometry
E&L: extractables and leachables
SVM: support vector machine
MRE: median relative errors
NIAS: non-intentionally added substance
FCM: food contact materials
IAS: intentionally added substances
SMLs: specific migration limits
GC–MS: gas chromatography–mass spectrometry
LC–MS: liquid chromatography–mass spectrometry
HRMS: high-resolution mass spectrometer
DIA: data independent acquisition
IMS: ion mobility spectrometry
UHPLC-IMS-MS: ultra-high performance liquid chromatography with an ion mobility-mass spectrometer
CCS: collision cross section
PLS-R: partial least squares regression
SVR: support vector regression
ANN: artificial neural network
MDs: molecular descriptors
MQNs: molecular quantum numbers
CNN: neural network model
SMILESs: simplified molecular-input line-entry systems
^TW^CCS_N2_: traveling wave collision cross section in nitrogen
ESI: electrospray ionization
CID: collision-induced dissociation
OCHEM: Online Chemical database
RMSECV: root mean squared error of cross validation
SIMPLS: statistically inspired modification of the partial least squares
*C*: cost of constraints violation
SR: sensitivity ratio
PA6: polyamide 6
PA66: polyamide 66
ATD: arrival time distributions
GB: gradient boosting
BHT: butylated hydroxytoluene
cIMS: cyclic ion mobility spectrometry
SLIM: structures for lossless ion manipulations
ML: machine learning
