# Supplementary material for: Prediction of Collision Cross Section Values: Application to Non-Intentionally Added Substance Identification in Food Contact Materials
Source: J Agric Food Chem. 2022 Jan 18;70(4):1272–81. doi: 10.1021/acs.jafc.1c06989 (PMC8815070; doi:10.1021/acs.jafc.1c06989)
Supplement: Supplementary file 1 — jf1c06989_si_001.pdf [file jf1c06989_si_001.pdf]

Supporting Information

Prediction of Collision Cross Section Values: Application to Non-Intentionally Added Substance (NIAS) Identification in Food Contact Materials

Xue-Chao Song[1,#], Nicola Dreolin[2,#], Tito Damiani[3,#], Elena Canellas[1], Cristina Nerin[1,*]

1. Department of Analytical Chemistry, Aragon Institute of Engineering Research I3A, CPS-University of Zaragoza, Maria de Luna 3, 50018, Zaragoza, Spain.

2. Waters Corporation, Altrincham Road, SK9 4AX, Wilmslow, United Kingdom.

3. Institute of Organic Chemistry and Biochemistry, Flemingovo náměstí 542/2, 160 00, Prague, Czech Republic.

* Corresponding author: Cristina Nerin, cnerin@unizar.es, Tel: +34 976761873.

Table of Contents:

Experimental Section: Molecular Modeling.

Table S1: Molecular formula, monoisotopic mass, retention time (RT) and $^{TW}CCS_{N2}$ of 9 compounds in our in-house made Test-Mix.

Table S2: Distribution of the molecular descriptors (MDs) calculated by alvaDesc software v.2.0.4.

Table S3: Descriptive statistical analysis of $^{TW}CCS_{N2}$ (Å^2) in calibration and validation sets.

Table S4. Validation indices of PLS and SVM model using AlvaDesc descriptors. R^2_p: external validation coefficient of determination; RMSEP: root mean square error of prediction; <2%, <3%, 5%: the proportions of molecules with predicted error less than 2%, 3% and 5%, respectively; MRE: median relative error.

Table S5. Predictive performance indices of the tested models for the prediction of extractables and leachables compounds analyzed in this study. R^2_p = external validation coefficient of determination; RMSEP = root mean square error of prediction; MRE = median relative error. <5% = percentage of predicted values below ±5% relative error; <2% = percentage of predicted values below ±2% relative error.

Table S6. Experimental and predicted $^{TW}CCS_{N2}$ of tentatively identified oligomers from adhesive and polyamides (PAs).

Figure S1. Distribution of data points in training and testing sets for $[M+H]^+$ and $[M+Na]^+$.
Figure S2. Histograms showing the distribution of original (A) and natural logarithm (B) of $^{TW}CCS_{N2}$ values for the protonated (top) and sodiated adducts (bottom).

Figure S3. Chemical classes of compounds for (A) $[M+H]^+$ and (B) $[M+Na]^+$.

Figure S4. Arrival time distribution (ATD) of N-Ethyl-p-toluenesulfonamide (m/z 199.0667) and its possible charge isomers in acetonitrile/water mobile phase.

Figure S5. Arrival time distribution (ATD) of 12-aminododecanolactam.

Figure S6. (A) Mass spectra of tributyl phosphate; (B) mobility trace of $[M+H]^+$; (C) mobility trace of $[M+Na]^+$; (D) possible structure of the dimer $[2M+Na]^+$ of tributyl phosphate; (E) drift time of $[M+H]^+$, $[M+Na]^+$ and dimeric ions.

Figure S7. Prediction performances of PLS and SVM before and after variable selection.

Figure S8. SR values of first 25 important molecular descriptors in $[M+H]^+$ and $[M+Na]^+$ models.

Figure S9. (A) Chromatogram of 1,6,13,18,25,30-hexaoxacyclohexatriacontane-7,12,19,24,31,36-hexone; (B) Low- (top) and high-energy spectra (bottom) of the detected compound, highlighting in green the molecular adducts (insert showing the experimental and predicted $^{TW}CCS_{N2}$, relative percentage deviation between brackets). Two common fragments are shown in the high-energy spectrum.

Figure S10. Sticks-and-balls 3-D structure of N-Ethyl-p-toluenesulfonamide charge isomers after geometry optimization with the DFTB3 method. PA = Proton Affinity.

Figure S11. Universal Force Field energy plot showing the different energy levels. Insert: 3D conformation of the lowest energy level and relative Coulomb potential in atomic units.

Results and Discussion: Molecular Modeling.

Experimental Section

Molecular Modeling. Geometry pre-optimization of the neutral molecule was performed using the Universal Force Field (UFF) model. Energy minimization of the neutral molecule was performed using the Amsterdam Density Functional engine (ADF),[1] where Density Functional (exchange-and-correlation functional, XC) was set as Local Density Approximation (LDA), and Slater Type Orbitals basis set was selected to be double zeta polarized basis (DZP).[2] LDA implies that the XC functional in each point in space depends only on the spin density in that same point. The UFF energy plot showing the different conformation levels was obtained using distance-geometry conformation generator by RDKit.[3]

The calculation of the total energy ($\boldsymbol{E}$) of the energetically refined neutral molecule and the $[M+H]^+$ adducts considering all protonation sites was performed using an extension of the Self-Consistent-Charge Density-Functional Tight-Binding Method (DFTB3). This is an approximate quantum chemical method derived from density functional theory (DFT) based on a second-order expansion of the DFT total energy around a reference density, and it features an improved Coulomb interaction between atomic partial charges and the complete third-order expansion of the DFT total energy.[4] The proton affinity ($\boldsymbol{PA}$) is computed as:

$$\boldsymbol{PA} = \boldsymbol{E}(\boldsymbol{B}) + \boldsymbol{E}(\boldsymbol{H}^+) - \boldsymbol{E}(\boldsymbol{BH}^+)$$

for a base ($\boldsymbol{B}$), where the total energy of the proton $\boldsymbol{E}(\boldsymbol{H}^+)$ was set to 0.24070 Hartree.

All operations and computations were performed using the Amsterdam Density Functional program and Density Functional Tight Binding engine (ADF & DFTB 2020, SCM, Theoretical Chemistry, Vrije University, Amsterdam, The Netherlands (http://www.scm.com).

Table S1. Molecular formula, monoisotopic mass, retention time (RT) and $^{TW}CCS_{N2}$ of 9 compounds in our *in-house* made Test-Mix.

Compound	Molecular formula	Monoisotopic mass (Da)	RT (min)	Expected CCS (Å^2)
Acetaminophen	$C_8H_9NO_2$	151.0633	1.96	131.5
Caffeine	$C_8H_{10}N_4O_2$	194.0804	2.76	138.2
Sulfaguanidine	$C_7H_{10}N_4O_2S$	214.0524	1.02	146.8
Sulfadimethoxine	$C_{12}H_{14}N_4O_4S$	310.0736	3.83	168.4
Val-tyr-val	$C_{19}H_{29}N_3O_5$	379.2107	2.87	191.7
Verapamil	$C_{27}H_{38}N_2O_4$	454.2832	4.57	208.8
Terfenadine	$C_{32}H_{41}NO_2$	471.3137	5.64	228.7
Leucine-enkephalin	$C_{28}H_{37}N_5O_7$	555.2693	3.97	229.8
Reserpine	$C_{33}H_{40}N_2O_9$	608.2734	4.92	252.3

Table S2. Distribution of the molecular descriptors (MDs) calculated by alvaDesc software v.2.0.4.

Blocks	Number of variables	Blocks	Number of variables
Constitutional descriptor	50	Ring descriptors	35
Topological indices	79	Walk and path counts	46
Connectivity indices	37	Information indices	51
2D-matrix-based descriptors	608	2D autocorrelations	213
Burden eigenvalues	96	P-VSA-like descriptors	69
ETA indices	40	Edge adjacency indices	324
Geometrical descriptors	38	3D matrix-based descriptors	132
3D autocorrelations	80	RDF descriptors	210
3D-MoRSE descriptors	224	WHIM descriptors	114
GETAWAY descriptors	273	Randic molecular profiles	41
Functional group counts	154	Atom centered fragments	115
Atom-type E-state indices	346	Pharmacophore descriptors	165
2D Atom Pairs	1596	3D Atom Pairs	36
Charge descriptors	15	Molecular properties	27
Drug-like indices	30	CATS 3D	300
WHALES	33	MDE	19
Chirality	70		

Table S3. Descriptive statistical analysis of ${}^{TW}CCS_{N2}$ (Å^2) in calibration and validation sets.

Adducts	Data set	Min	Max	Mean	Median	Range	SD
[M+H]	Calibration	118.6	329.4	167.5	159.6	210.9	35.4
	Validation	121.3	283.4	161.2	152.2	162.2	36.0
[M+Na]	Calibration	136.8	311.4	191.7	186.9	174.6	32.3
	Validation	142.6	287.9	191.4	184.0	145.3	36.0

Table S4. Validation indices of PLS and SVM model using AlvaDesc descriptors. R^2_p: external validation coefficient of determination; RMSEP: root mean square error of prediction; <2%, <3%, 5%: the proportions of molecules with predicted error less than 2%, 3% and 5%, respectively; MRE: median relative error.

Adducts	Descriptors	Algorithm	R^2_P	RMSEP	<2%	<3%	<5%	MRE
$[M+H]^+$	alvaDesc_3036	SIMPLS	0.980	5.3	58.9%	70.5%	93.7%	1.59%
		KernelPLS	0.983	4.7	57.9%	72.6%	93.7%	1.44%
		**SVM**	0.984	4.5	62.1%	74.7%	92.6%	1.50%
	alvaDesc_1029	SIMPLS	0.986	4.3	60.0%	71.6%	95.8%	1.81%
		SVM	0.987	4.2	53.7%	71.6%	96.8%	1.77%
$[M+Na]^+$	alvaDesc_3036	SIMPLS	0.949	8.0	45.3%	62.5%	82.8%	2.16%
		KernelPLS	0.949	8.1	45.3%	64.1%	81.3%	2.14%
		**SVM**	0.948	8.2	54.7%	71.9%	81.3%	1.82%
	alvaDesc_862	SIMPLS	0.922	10.0	40.6%	51.6%	76.6%	2.81%
		SVM	0.926	9.6	48.4%	64.0%	79.7%	2.18%

Table S5. Predictive performance indices of the tested models for the prediction of extractables and leachables compounds analyzed in this study. R^2_p = external validation coefficient of determination; RMSEP = root mean square error of prediction; MRE = median relative error. <5% = percentage of predicted values below ±5% relative error; <2% = percentage of predicted values below ±2% relative error.

Adducts	Models	R^2_p	RMSEP	<2%	<5%	MRE
M+H	SVM	0.984	4.5	62.1%	92.6%	1.50%
	CCSondemand	0.986	4.3	62.5%	92.6%	1.35%
	AllCCS	0.971	6.6	41.1%	83.2%	2.41%
	CCSbase	0.976	5.9	48.4%	81.1%	2.02%
M+Na	SVM	0.948	8.2	54.7%	81.3%	1.82%
	CCSondemand	0.949	8.9	34.4%	76.6%	1.82%
	AllCCS	0.922	13.3	25.0%	54.7%	4.37%
	CCSbase	0.940	8.8	37.5%	71.9%	2.42%

Table S6. Experimental and predicted $^{TW}CCS_{N2}$ of tentatively identified oligomers from adhesive and polyamides (PAs).

	Compounds	Neutral Monoisotopic Mass	Adducts	Experimental $^{TW}CCS_{N2}$ (Å^2)	Predicted $^{TW}CCS_{N2}$ (Å^2)	Deviation (%)
Adhesive oligomers	1,6-Dioxacyclododecane-7,12-dione	200.1049	$[M+H]^+$	-	-	-
			$[M+Na]^+$	148.6	154.4	3.9
	1,6,13,18-Tetraoxacyclotetracosane-7,12,19,24-tetrone	400.2097	$[M+H]^+$	189.2	194.8	3.0
			$[M+Na]^+$	188.4	193.2	2.6
	1,6,13,18,25,30-Hexaoxacyclohexatriacontane-7,12,19,24,31,36-hexone	600.3146	$[M+H]^+$	234.2	234.9	0.3
			$[M+Na]^+$	228.5	234.8	2.8
	1,6,13,18,25,30,37,42-Octaoxacyclooctatetracontane-7,12,19,24,31,36,43,48-octaone	800.4194	$[M+H]^+$	275.1	262.9	**-4.4**
			$[M+Na]^+$	266.9	261.2	-2.1
PA 6 oligomers	1,8-Diazacyclotetradecane-2,9-dione	226.1681	$[M+H]^+$	152.5	152.1	-0.2
			$[M+Na]^+$	155.5	163.0	**4.9**
	1,8,15-Triazacyclohenicosane-2,9,16-trione	339.2522	$[M+H]^+$	180.0	177.9	-1.2
			$[M+Na]^+$	181.6	185.8	2.3
	1,8,15,22-Tetraazacyclooctacosane-2,9,16,23-tetrone	452.3363	$[M+H]^+$	208.0	208.1	0.1
			$[M+Na]^+$	212.7	214.6	0.9
	1,8,15,22,29-Pentazacyclopentatriacontane-2,9,16,23,30-pentone	565.4203	$[M+H]^+$	235.5	231.7	-1.6
			$[M+Na]^+$	232.6	234.8	1.0
	1,8,15,22,29,36-Hexazacyclodotetracontane-2,9,16,23,30,37-hexone	678.5044	$[M+H]^+$	261.9	259.7	-0.8
			$[M+Na]^+$	261.5	259.7	-0.7
PA 66 oligomers	1,8-Diazacyclotetradecane-2,7-dione	226.1681	$[M+H]^+$	151.3	150.6	-0.4
			$[M+Na]^+$	155.7	163.4	**5.0**
	1,8,15,22-Tetraazacyclooctacosane-2,7,16,21-tetrone	452.3363	$[M+H]^+$	210.6	210.9	0.2
			$[M+Na]^+$	213.2	216.4	1.5
	1,8,15,22,29,36-Hexaazacyclodotetracontane-2,7,16,21,30,35-hexone	678.5044	$[M+H]^+$	269.4	258.2	**-4.2**
			$[M+Na]^+$	266.9	262.8	-1.6

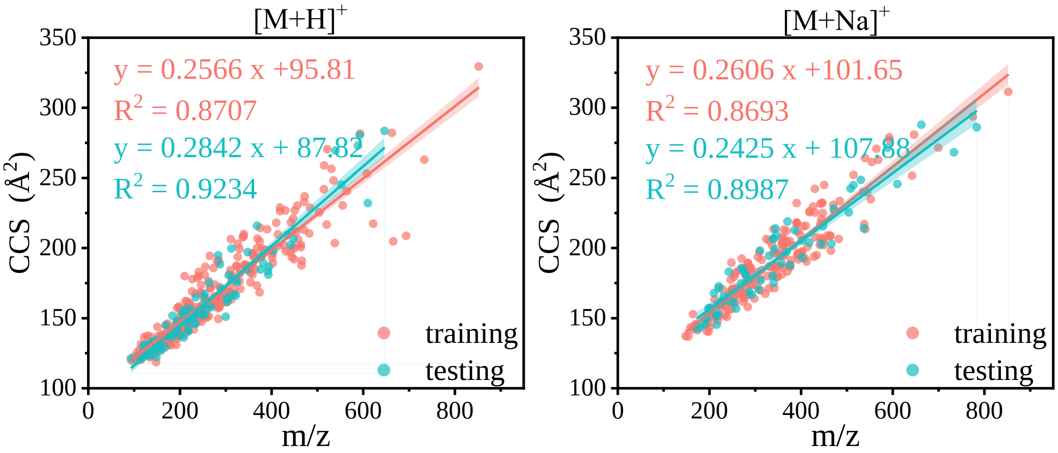

Figure S1. Distribution of data points in training and testing sets for $[M+H]^+$ and $[M+Na]^+$.

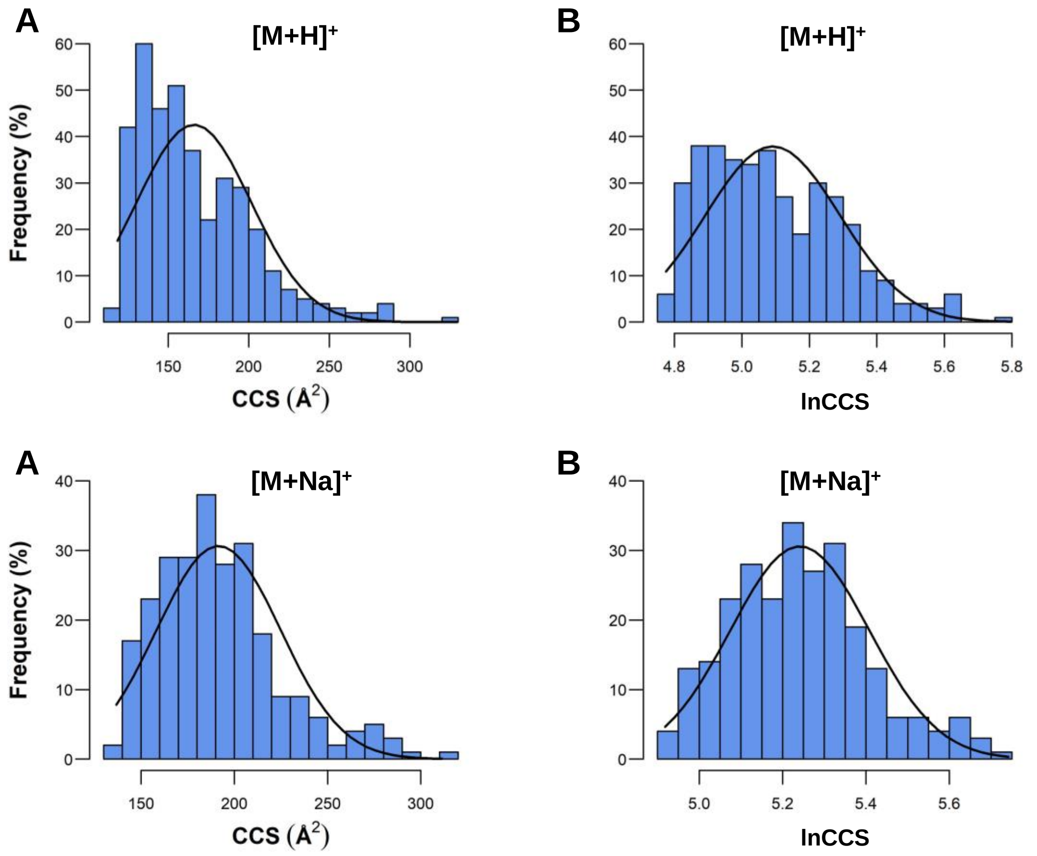

Figure S2. Histograms showing the distribution of original (**A**) and natural logarithm (**B**) of $^{TW}CCS_{N2}$ values for the protonated (top) and sodiated adducts (bottom).

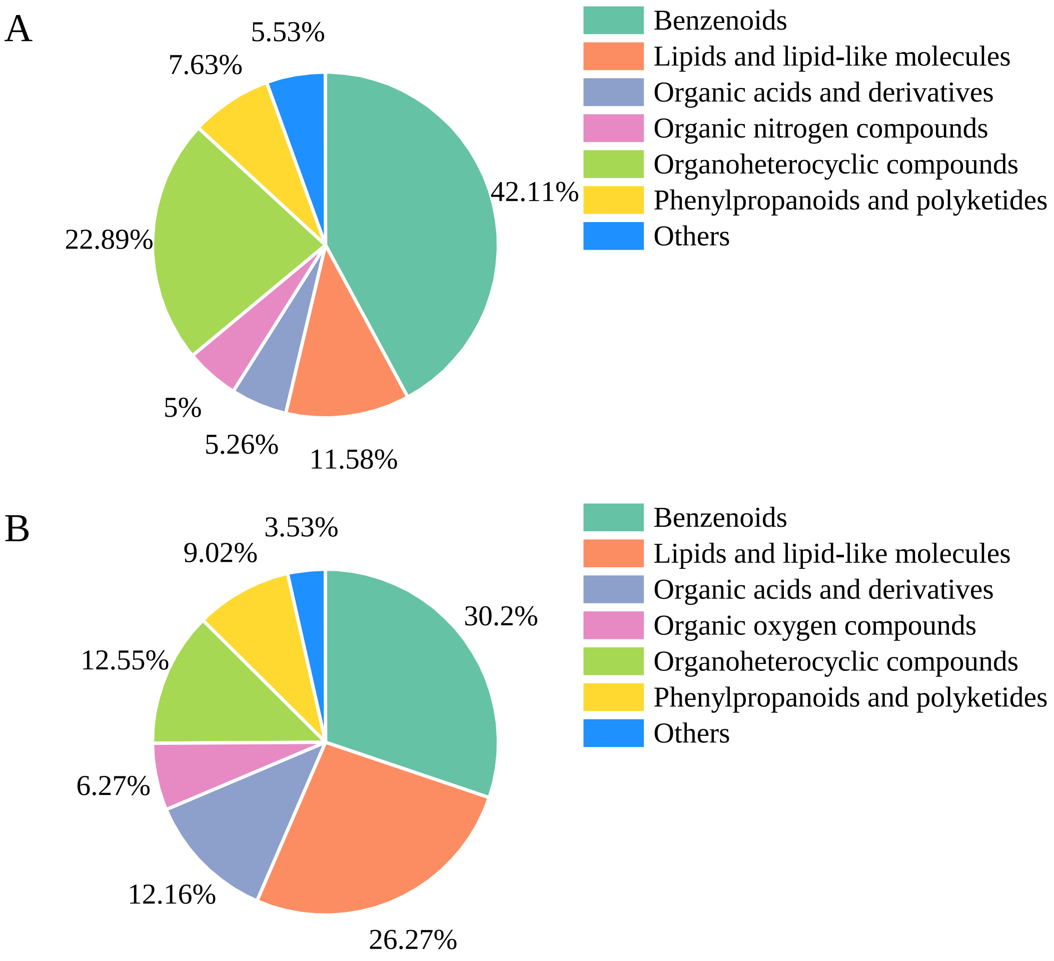

Figure S3. Chemical classes of compounds for (A) $[M+H]^+$ and (B) $[M+Na]^+$.

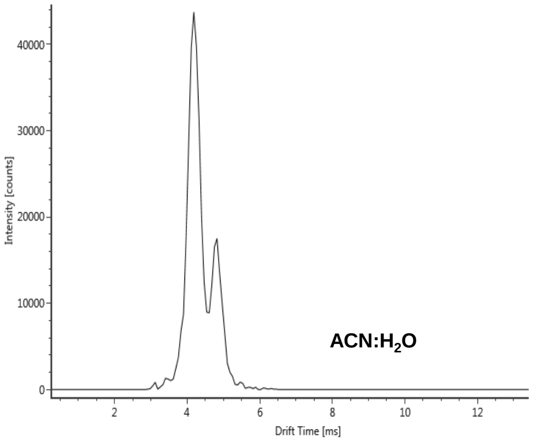

Figure S4. Arrival time distribution (ATD) of N-Ethyl-p-toluenesulfonamide (*m/z* 199.0667) and its possible charge isomers in acetonitrile/water mobile phase.

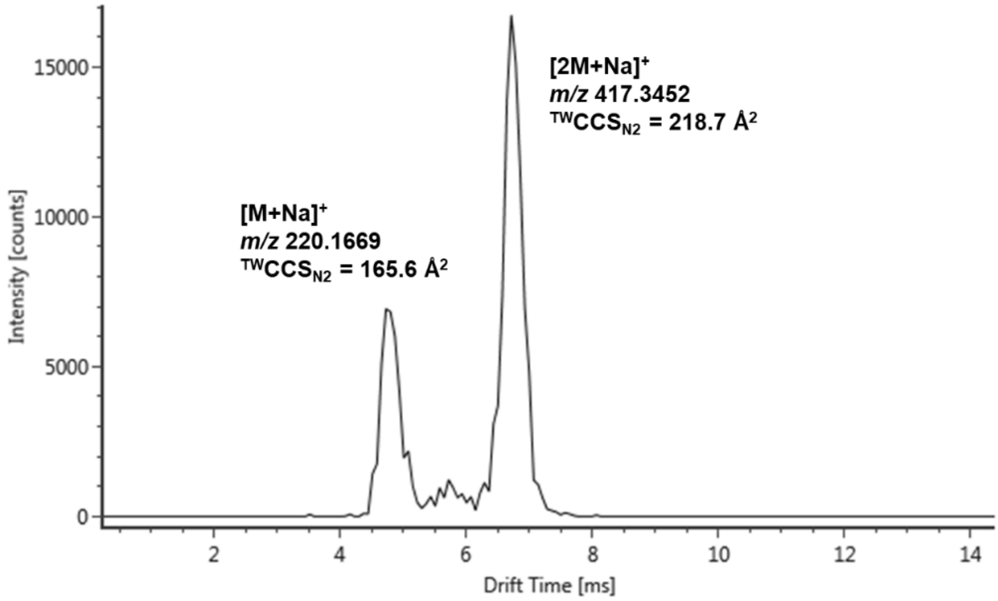

Figure S5. Arrival time distribution (ATD) of 12-aminododecanolactam.

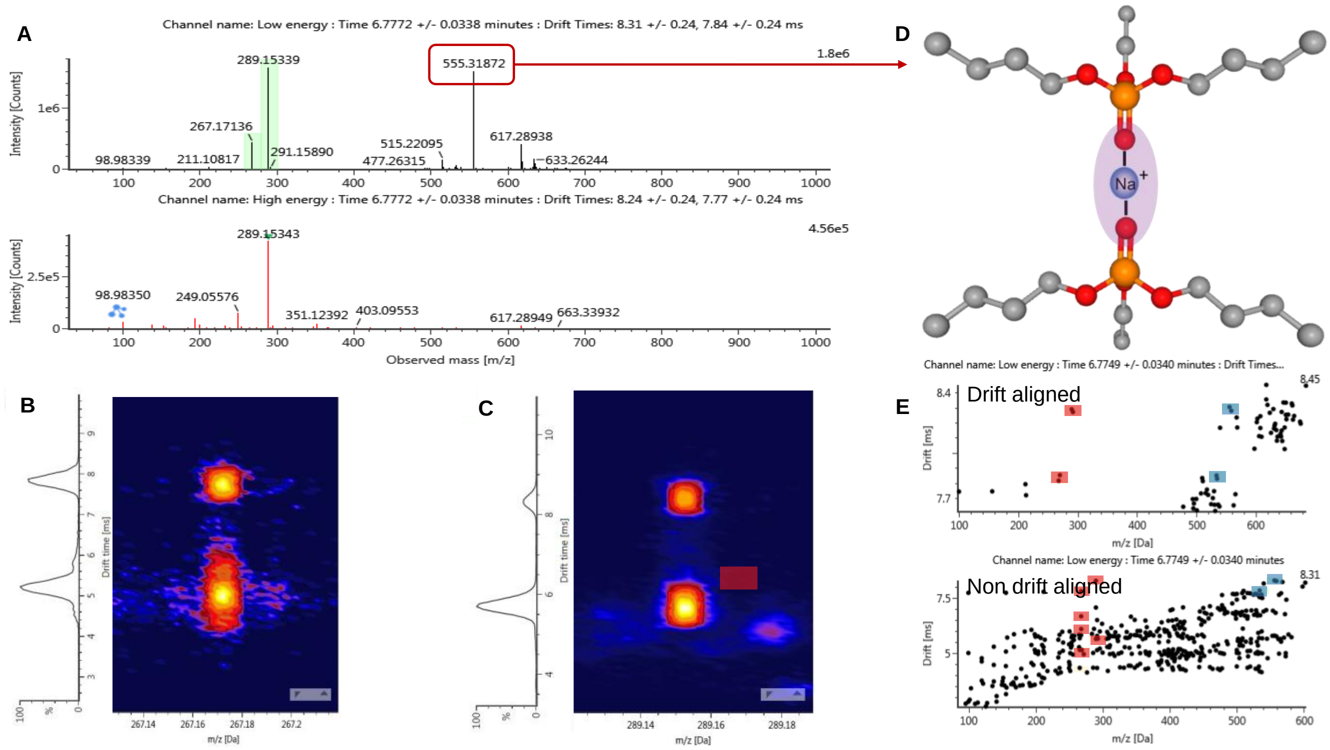

Figure S6. (**A**) Mass spectra of tributyl phosphate; (**B**) mobility trace of $[M+H]^+$; (**C**) mobility trace of $[M+Na]^+$; (**D**) possible structure of the dimer $[2M+Na]^+$ of tributyl phosphate; (**E**) drift time of $[M+H]^+$, $[M+Na]^+$ and dimeric ions.

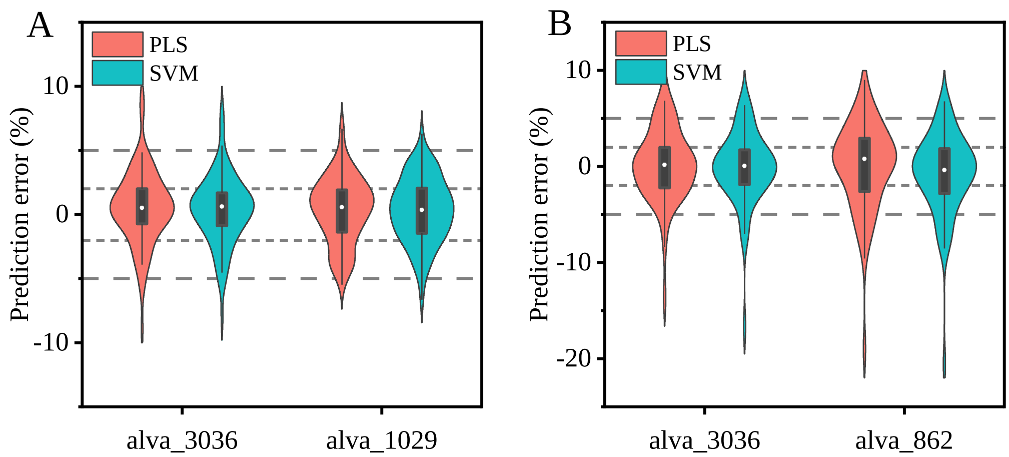

Figure S7. Prediction performances of PLS and SVM before and after variable selection.

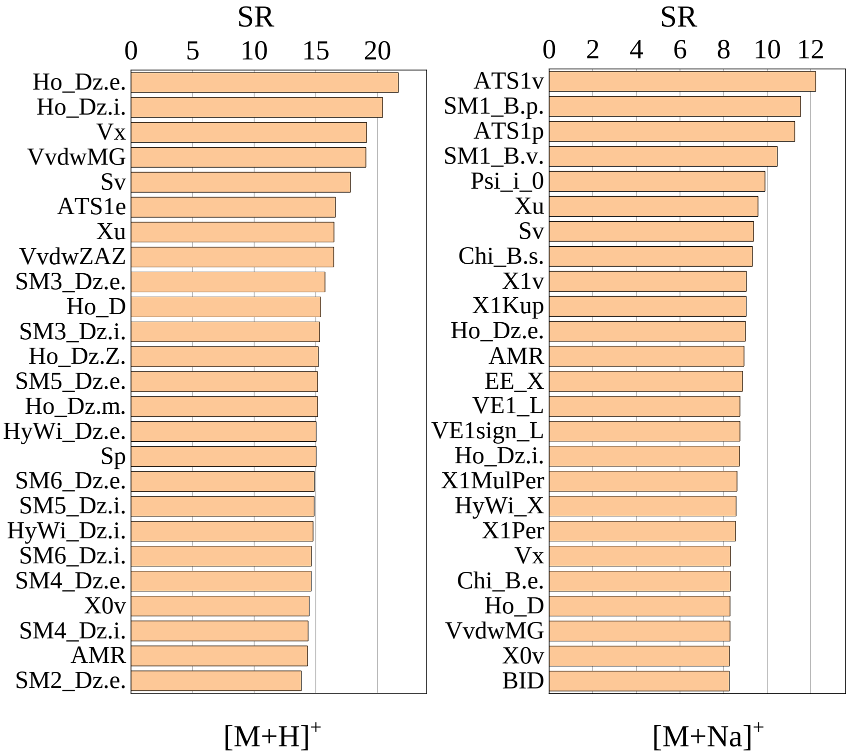

Figure S8. SR values of first 25 important molecular descriptors in $[M+H]^+$ and $[M+Na]^+$ models.

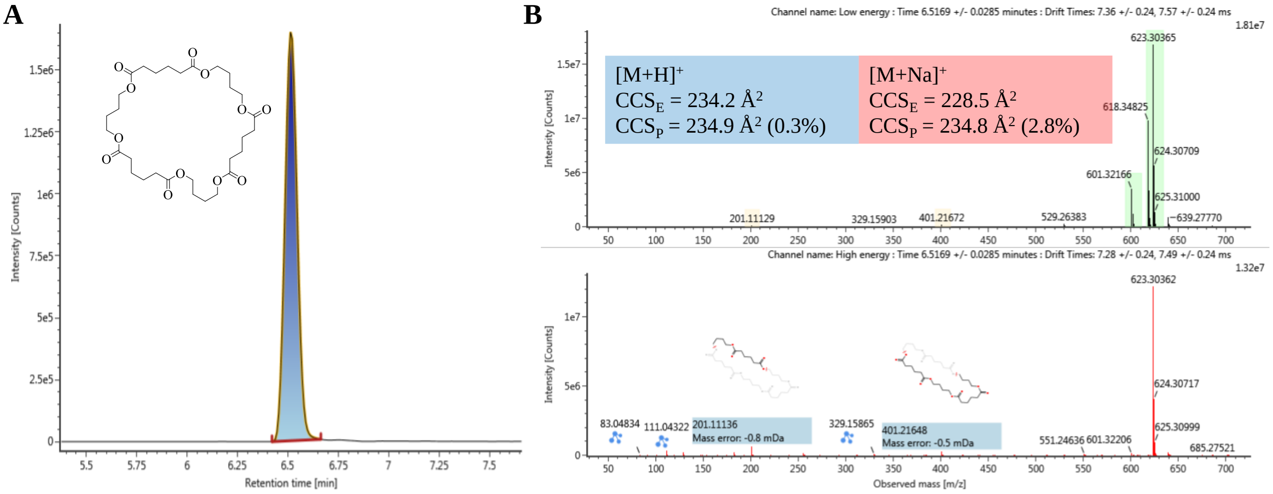

Figure S9. (**A**) Chromatogram of 1,6,13,18,25,30-hexaoxacyclohexatriacontane-7,12,19,24,31,36-hexone; (**B**) Low- (top) and high-energy spectra (bottom) of the detected compound, highlighting in green the molecular adducts (insert showing the experimental and predicted $^{TW}CCS_{N2}$, relative percentage deviation between brackets). Two common fragments are shown in the high-energy spectrum.

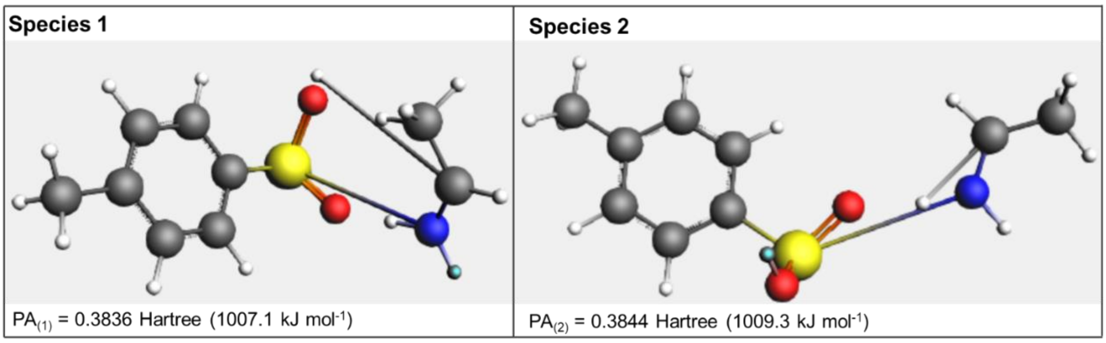

Figure S10. Sticks-and-balls 3-D structure of N-Ethyl-p-toluenesulfonamide charge isomers after geometry optimization with the DFTB3 method. PA = Proton Affinity.

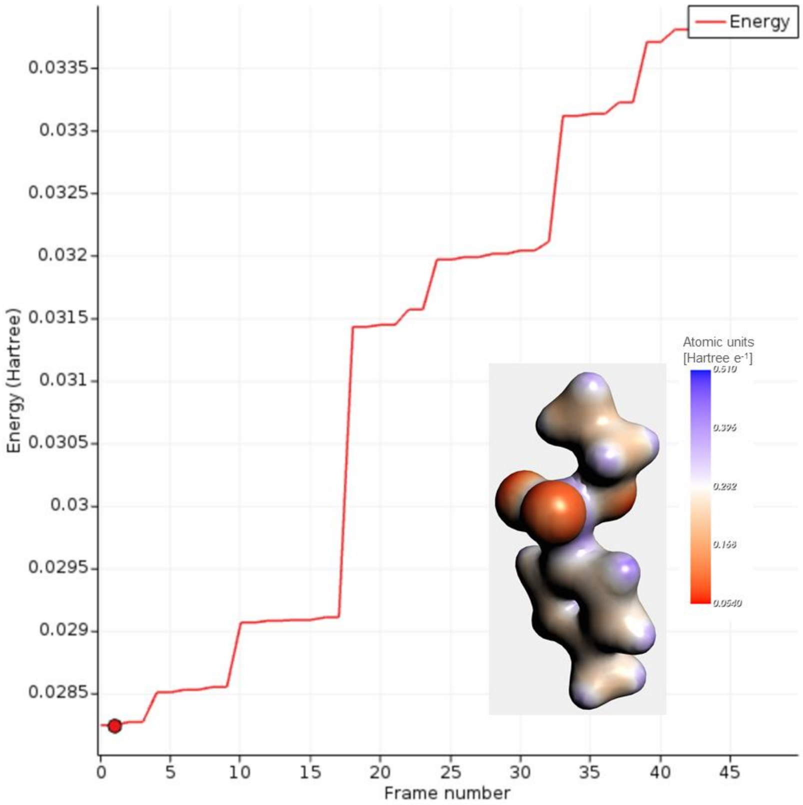

Figure S11. Universal Force Field energy plot showing the different energy levels. Insert: 3D conformation of the lowest energy level and relative Coulomb potential in atomic units.

Results and Discussion

Molecular Modeling. Modelling To better understand whether the presence of two charge isomers represents a plausible explanation to the experimental findings, we computed and compared the proton affinity (PA), defined as the negative gas phase enthalpy for the protonation reaction $B + H^+ \rightarrow BH^+$. After energy minimization of the neutral molecule, we considered the above-mentioned electronegativity centers (the two sulfonamide oxygens were considered equivalent) using the approximate quantum chemical method DFTB3.[4] The resulting PAs were found to be comparable (1007.1 and 1009.3 kJ mol^{-1} for the species 1 and 2, respectively), thus confirming the presence of two equivalent protonation sites on the molecule. Figure S10 shows the 3D chemical structure of the isomers after geometry optimization, where the additional proton is marked by a light-blue dot. A similar conclusion could be inferred by the visual interpretation of the coulombic potential of the neutral molecule (insert of Figure S11), where the red regions represent those with higher electron density, thus the more probable location for protonation. Also, it is reasonable to think of species #2 to possess a higher collision cross section as the $O\text{-}H^+$ protrudes from the molecular core, increasing the rotationally averaged size of the ion, which in turn is translated to a higher drift time compared to species #1. Notably, the two isomers presented a difference of 16.5 $Å^2$, which enabled their separation in the ion mobility dimension.

(1). te Velde, G.; Bickelhaupt, F. M.; Baerends, E. J.; Fonseca Guerra, C.; van Gisbergen, S. J. A.; Snijders, J. G.; Ziegler, T., Chemistry with ADF. *J. Comput. Chem.* **2001,** *22,* (9), 931-967.
(2). Van Lenthe, E.; Baerends, E. J., Optimized Slater-type basis sets for the elements 1–118. *J. Comput. Chem.* **2003,** *24*, (9), 1142-1156.
(3). Landrum, G. RDKit: Open-Source Cheminformatics Software. http://www.rdkit.org/.(accessed Feb 1, 2021).
(4). Gaus, M.; Cui, Q.; Elstner, M., DFTB3: Extension of the self-consistent-charge density-functional tight-binding method (SCC-DFTB). *J. Chem. Theory Comput.* **2012,** *7*, (4), 931-948.
